# Supplementary material for: Complex‐centric proteome profiling by SEC‐SWATH‐MS
Source: Mol Syst Biol. 2019 Jan 14;15(1):e8438. doi: 10.15252/msb.20188438 (PMC6346213; doi:10.15252/msb.20188438)
Supplement: Supplementary file 8 — Dataset EV7 [file MSB-15-e8438-s008.zip › feature_plots_string/P05165.pdf]

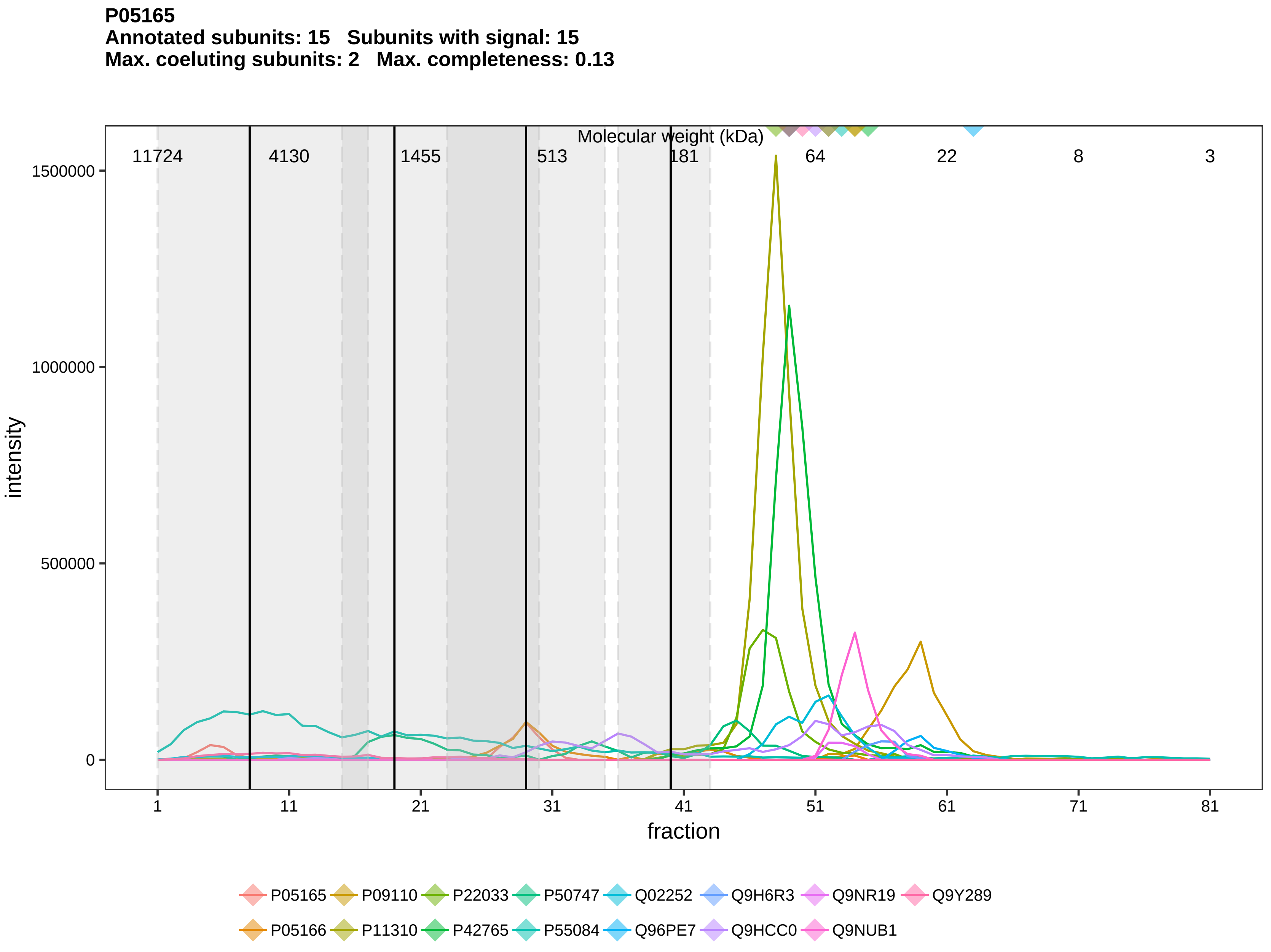

P05165
Annotated subunits: 15 Subunits with signal: 15
Max. coeluting subunits: 2 Max. completeness: 0.13
Molecular weight (kDa)
11724
4130
1455
513
181
64
22
8
3
intensity
1500000
1000000
500000
0
fraction
1
11
21
31
41
51
61
71
81
P05165
P09110
P22033
P50747
Q02252
Q9H6R3
Q9NR19
Q9Y289
P05166
P11310
P42765
P55084
Q96PE7
Q9HCC0
Q9NUB1
